# Molecular Insight into the Steric Shielding Effect of PEG on the Conjugated Staphylokinase: Biochemical Characterization and Molecular Dynamics Simulation

**DOI:** 10.1371/journal.pone.0068559

**Published:** 2013-07-18

**Authors:** Qimeng Mu, Tao Hu, Jingkai Yu

**Affiliations:** 1 National Key Laboratory of Biochemical Engineering, Institute of Process Engineering, Chinese Academy of Sciences, Beijing, China; 2 University of Chinese Academy of Sciences, Beijing, China; Bioinformatics Institute, Singapore

## Abstract

PEGylation is a successful approach to improve potency of a therapeutic protein. The improved therapeutic potency is mainly due to the steric shielding effect of PEG. However, the underlying mechanism of this effect on the protein is not well understood, especially on the protein interaction with its high molecular weight substrate or receptor. Here, experimental study and molecular dynamics simulation were used to provide molecular insight into the interaction between the PEGylated protein and its receptor. Staphylokinase (Sak), a therapeutic protein for coronary thrombolysis, was used as a model protein. Four PEGylated Saks were prepared by site-specific conjugation of 5 kDa/20 kDa PEG to N-terminus and C-terminus of Sak, respectively. Experimental study suggests that the native conformation of Sak is essentially not altered by PEGylation. In contrast, the bioactivity, the hydrodynamic volume and the molecular symmetric shape of the PEGylated Sak are altered and dependent on the PEG chain length and the PEGylation site. Molecular modeling of the PEGylated Saks suggests that the PEG chain remains highly flexible and can form a distinctive hydrated layer, thereby resulting in the steric shielding effect of PEG. Docking analyses indicate that the binding affinity of Sak to its receptor is dependent on the PEG chain length and the PEGylation site. Computational simulation results explain experimental data well. Our present study clarifies molecular details of PEG chain on protein surface and may be essential to the rational design, fabrication and clinical application of PEGylated proteins.

## Introduction

PEGylation, conjugation of polyethylene glycol (PEG) to a therapeutic protein, is a successful approach to improve therapeutic potency of proteins [1], [2]. The therapeutic potency is improved by increasing serum half-life, decreasing immunogenicity, and reducing renal clearance and proteolytic degradation of the protein [3]–[5]. Typically, a therapeutic protein is conjugated with PEG via a covalent linkage at some reactive moieties of the protein. PEG entangles around the protein surface through hydrophobic interaction and concurrently forms hydrogen bonds with the surrounding water molecules. On one hand, the steric shielding effect of PEG can achieve the advantages mentioned above. On the other hand, PEG may sterically shield the bioactive domain (e.g., substrate or receptor binding domain) of a protein, resulting in a substantial loss of its bioactivity [4]–[6]. Therefore, it is of interest to understand the steric shielding effect of PEG on the interaction between a protein and its substrate/receptor.

The PEGylation site and the PEG mass are two main factors that influence the bioactivity of the protein. Site-specific PEGylation of a protein far from its bioactive domain has been demonstrated to decrease the loss of bioactivity of the PEGylated protein [7], [8]. In contrast, PEGylation at the bioactive domain may result in complete loss of bioactivity of a protein. However, this approach can not completely avoid the loss of bioactivity, due to the presence of the steric shielding effect of PEG [9], [10]. Some therapeutic proteins, whose pharmacological effects involve high molecular weight (M_w_) substrate or receptor interactions, show *in vitro* bioactivity is inversely proportional to the mass of attached PEG. For example, Chiu *et al*. prepared PEGylated trypsin conjugated with 2, 5, 10 and 20 kDa PEG, respectively [11]. PEG with 10 kDa was demonstrated to be the optimal size to improve thermal stability and maintain bioactivity under physiological conditions. However, the molecular mechanism of the interaction between the PEGylated protein and its receptor is not adequately clear.

Molecular dynamics simulation is an effective approach to reveal atomic level details of biological interactions [12], [13] and has been used for PEG-protein simulation. For example, Manjula *et al*. built the PEG-hemoglobin models by means of molecular dynamics simulation [14]. They found that the PEG chain was folded loosely on the surface of hemoglobin and the coverage was not proportional to the PEG chain length. Yang *et al*. modeled the PEG-insulin system and found the optimized conformations of the conjugates using a simulated annealing method [15]. However, these studies focus on the effects of the PEG chain length on the properties of PEGylated protein, where the effect of the PEGylation sites is ignored. In addition, the underlying mechanism of the steric shielding effect of PEG on the protein therapeutic potency is not well understood, especially for protein interaction with its high M_w_ substrate or receptor.

**Figure 1:**
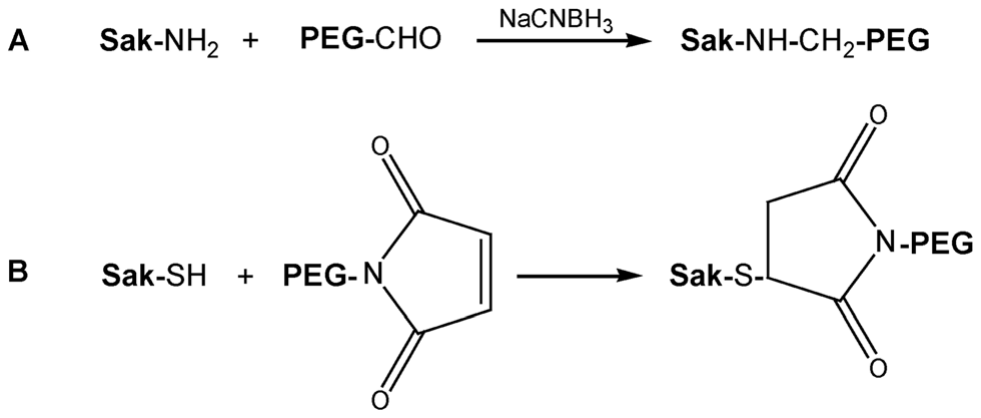
Schematic presentation of the PEGylated reaction. (A) PEGylation at N-terminus of Sak. (B) PEGylation at C-terminus of Sak.

In the present work, experimental studies and molecular dynamics simulations were used to provide a molecular insight into the interaction between the PEGylated protein and its receptor at the atomic level. Staphylokinase (Sak) is a bioactive protein that can disintegrate thrombus through activating the plasma plasminogen to plasmin and is used as the model protein here [16], [17]. Compared with native Sak, Sak used here lacks 10 amino acid residues at the N-terminus and has an additional peptide of Gly-Gly-Cys at the C-terminus [18]. In this study, 5 kDa and 20 kDa PEG aldehydes were used for site-specific PEGylation of Sak at the α-amino group at the N-terminus (Fig. 1A). In addition, 5 kDa and 20 kDa PEG maleimides were used for site-specific PEGylation of Sak at the thiol group at the C-terminus (Fig. 1B). The structural and functional properties of the four PEGylated Saks were characterized and compared with the free Sak.

Molecular models of the PEGylated Saks were constructed and molecular dynamics simulations of the PEGylated Saks performed. Docking analyses were carried out to investigate interaction between the PEGylated Sak and its receptor, plasminogen. Plasminogen was represented by micro-plasminogen (micro-plg) for docking purpose. Molecular dynamics simulations and docking analyses explain well the experimental results. Our study is expected to facilitate understanding of the steric shielding effect of PEG on a protein, especially the ones involving interactions with their high M_w_ substrate or receptor.

## Materials and Methods

### Materials

The recombinant staphylokinase (Sak) was produced in a transformed *E. coli* and purified by ion exchange chromatography and size exclusion chromatography as described previously [18]. Methoxyl PEG propionaldehyde of 5 kDa (ald5k) and 20 kDa (ald20k), methoxyl PEG maleimide with 5 kDa (mal5k) and methoxyl PEG maleimide with 20 kDa (mal20k) were purchased from Jenkem Biotech (Beijing, China). Tris(2-carboxyethyl)phosphine (TCEP) was obtained from Pierce (USA). NaCNBH_3_ was obtained from Sigma (USA).

### Preparation of the PEGylated Sak

#### C-terminally PEGylated Sak

Sak was incubated with TCEP at the Sak/TCEP molar ratio of 1∶10 in 20 mM sodium phosphate buffer, pH 7.2 (PB buffer). The incubation was conducted at room temperature for 3 h to prevent the S-S dimerization of Sak. Then, TCEP was removed by centrifugation at 5000 rpm with 3 kDa cutoff membrane against PB buffer for three times. Sak (1.5 mg/ml, 0.1 mM) in PB buffer was incubated with 0.4 mM mal5k and 0.4 mM mal20k at 4^o^C overnight, respectively.

#### N-terminally PEGylated Sak

TCEP treated Sak was dialyzed against 20 mM sodium acetate buffer (pH 5.0). Afterwards, Sak (0.1 mM) was incubated with 0.5 mM ald5k and 0.5 mM ald20k in the presence of 10 mM NaCNBH_3_, respectively. The incubation was in 20 mM sodium acetate buffer (pH 5.0) at 4^o^C overnight.

#### Purification of the PEGylated Saks

The reaction mixtures were subjected to an SP Sepharose HP column (1.6 cm×25 cm, GE Healthcare, USA) that was equilibrated with 20 mM NaAc-HAc buffer (pH 5.0, Buffer A). As for mal5k and mal20k treated Sak, the column was eluted with Buffer A for removal of the free PEG reagent, followed by elution with a linear salt gradient of 0–0.2 M NaCl in Buffer A for 30 min at a flow rate of 1.0 ml/min. The effluent was detected at 254 nm. The fractions corresponding to Sak-mal5k and Sak-mal-20k were pooled and concentrated for further experiment, respectively.

As for ald5k and ald20k treated Sak, the column was eluted with 12 ml of Buffer A for removal of the free PEG reagent, followed by elution with 18 ml of 0.5 M NaCl in Buffer A at a flow rate of 1.0 ml/min. The effluent was detected at 254 nm. The fractions corresponding to the mixture of Sak and PEGylated Sak were pooled, respectively. The fractions were loaded on a Superdex 200 column (2.6 cm ×60 cm, GE Healthcare, USA) based on size exclusion chromatography (SEC). The column was equilibrated and eluted by 20 mM phosphate buffer (pH 7.2) at a flow rate of 3.0 ml/min. The fractions corresponding to Sak-ald5k and Sak-ald-20k were pooled and concentrated for further experiment.

### Analysis of PEGylated Sak

SEC analysis of the Sak samples was carried out at room temperature on a Superdex 200 column (1 cm×30 cm, GE Healthcare, USA). The column was equilibrated and eluted by PB buffer at a flow rate of 0.5 ml/min. The effluent was detected at 280 nm. SDS-PAGE analysis was performed on a 14% polyacrylamide gel. The gel was stained with Coomassie blue R-250.

### Circular dichroism spectroscopy

Circular dichroism (CD) spectra of the products were recorded on a Jasco-810 spectropolarimeter (Jasco, Japan) from 260 to 195 nm at the room temperature. A cuvette with 0.2 cm pathlength was used. All samples were at a Sak concentration of 0.2 mg/ml in PB buffer.

### Fluorescence measurement

Intrinsic fluorescence measurements were performed using Hitachi F-4500 fluorescence spectrometer with a cuvette of 1 cm pathlength. The emission spectra were excited at 280 nm and recorded from 300 to 400 nm. Excitation and emission slit widths were both 5 nm. All samples were at a protein concentration of 0.1 mg/ml in PB buffer.

### Molecular radius measurement

Molecular radii were measured on a DynaPro Titan TC instrument (Wyatt, USA) at 25°C using the dynamic light scattering (DLS) method. The products were at the protein concentration of 1 mg/ml in PB buffer. The samples were centrifuged at 12,000 g for 10 min before analysis.

### Analytical ultracentrifugation

Sedimentation velocity was measured by analytical ultracentrifugation (AUC) on a ProteomeLab XL21 (Beckman, USA) equipped with an An260Ti rotor. The samples at the nominal concentration (A280 = 0.6) were centrifuged at 60,000 rpm in PB buffer. The 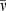 values (the partial specific volume) of 0.74 ml/g for Sak and 0.806 ml/g for the PEGylated proteins were used to calculate the sedimentation coefficients. The coefficients *S_20,w_* (the sedimentation coefficient) were normalized to standard conditions by correcting the buffer density and viscosity.

### *In vitro* biological activity

The *in vitro* biological activity of the products was tested by fibrin plate assay [18]. Ten ml of 1.5% (w/v) agarose was mixed with 10 ml of 0.5% (w/v) fibrinogen containing 10 U of thrombin in each petri dish. The buffer used was 50 mM Tris-HCl buffer containing 150 mM NaCl (pH 7.5). Holes with equal diameter were punched on the solidified agarose plate. Ten μl Sak samples at a protein concentration of 0.5 mg/ml were added to the holes and incubated at 37°C for 20 h. The diameters of the limpid areas around the holes represented biological activities of the samples, where the activity of unmodified Sak was regarded as 100%.

### Molecular dynamics simulation

The structure of Sak was obtained from the Protein Data Bank (1SSN) [19]. 1SSN is a collection of 20 NMR structures. RMSDs between all pairs of the 20 structures were computed and were all found to be less than 2Å. The first model was chosen for simulation. The first ten amino acids at the N-terminus were cut off and Met residue was then added. A segment containing three amino acid residues (Gly-Gly-Cys) was added to the C-terminus. PEGs with M_w_ of 5 kDa and 20 kDa were modeled in ArgusLab 4.0.1 [20]. For N-terminal PEGylation, PEG chain was linked to Met^1^ of Sak through a propyl moiety. For C-terminal PEGylation, PEG chain was linked to Cys^130^ of Sak (i.e., C-terminus) via a succimidyl moiety.

Molecular dynamics simulations were performed in GROMACS 4.5.4 [21] with GROMOS96 53a6 force field [22]. PEG parameterization was performed with quantum computation within Gaussian 09W [23], resultant parameters were integrated to the 53a6 force field to form a new force field, named 53a6_PEG. Its suitability for PEG simulation was validated by comparing its performance in PEG simulation to that of force field 53a6_OE developed in [24]. Figures S1 and S2 show that 53a6_PEG results in more extended PEG chains than 53a6_OE. Figures S3 and S4 show that radii of gyration (Rg) of PEG chains based on 53a6_PEG are larger than those based on 53a6_OE. Figures S1-S4 thus show that 53a6_PEG is better able to represent PEG's good solubility in water (see File S1 for details). Simulation visualization was performed in Visual Molecular Dynamics (VMD) from the theoretical and computational biophysics group at UIUC [25]. To evaluate initial PEG conformation's effect on simulation results, we had performed test simulation runs using PEGs with different initial conformations. Fig. S5A shows that the system attains a similar size starting from different initial PEG conformations. Fig. S5B shows that differences between final structures resulted from different initial conformations are small, all less than 1 nm. Figure S5 therefore shows that initial PEG conformation does not have much influence on its final wrapping state over the protein surface. Repeated MD trajectories were thus not generated in this work.

The four PEGylated Saks were solvated by the simple point charge (SPC) water model and neutralized by adding sodium ions (Na^+^). Triclinic cell was used for periodic boundary conditions. The van der Waals and electrostatic interactions were both switched at 0.9 nm and then cut off at 1.4 nm. Particle mesh Ewald (PME) method was employed for electrostatic interactions. Time step was set to 2 fs. Energy minimization was run first to reduce inappropriate solute-solvent contacts, followed by 100 ps NVT and 100 ps NPT equilibrations; after which a simulated annealing procedure with a NVT ensemble was performed. Temperature was controlled by velocity rescaling method. Steepest descent method was used for energy minimization. Simulated annealing was adopted to speed up simulation. Temperature was linearly increased from 300 to 400 K within 3 ns and then kept constant at 400 K for 2 ns. Temperature was further linearly increased to 450 K within 3 ns and kept constant at 450 K for another 2 ns, followed by a symmetric decrease to 300 K. The system was then kept at 300 K for additional 7 ns.

### Docking analyses

Configuration of PEGylated Sak at the end of simulation process was docked to micro-plg in HEX 6.12. Crystal structure of micro-plg was obtained from the Protein Data Bank (1QRZ) [26]. The centroids of PEGylated Sak and micro-plg were set within 30 Å of each other, which is the best working range for HEX docking [27]. The known crystal structure of micro-plg-Sak-micro-plg complex (1BUI) [28] provides Sak and micro-plg binding domains and their orientation toward each other, that information is used to set up initial positions of PEGylated Sak and micro-plg for HEX docking. Actual HEX docking parameters are listed in Table 1.

**Table 1:** Parameters used for HEX docking.

Parameter	Value	Meaning of the Parameter
Correlation type	Shape+Electrostatics	Functions to calculate the docking correlation between two molecules
FFT Mode	3D	Three-dimensional (3D) rotational correlation, for accelerating the rotation part of the search
Grid Dimension	0.6	The sampling grid size
Receptor range	30	The range angle of scanning at the receptor’s surface
Ligand Range	30	The range angle of scanning at the ligand's surface
Twist range	45	The intermolecular twist angle
Distance Range	40	The limit of intermolecular separation from the initial distance
Scan step	0.8	The scan unit of the distance
Steric Scan	20	The spherical polar docking expansion order (N) used for calculate electrostatic potential at the initial scan
Final Scan	25	The higher N used for final scan to refine the search

## Results

### Characterization of the products

**Figure 2:**
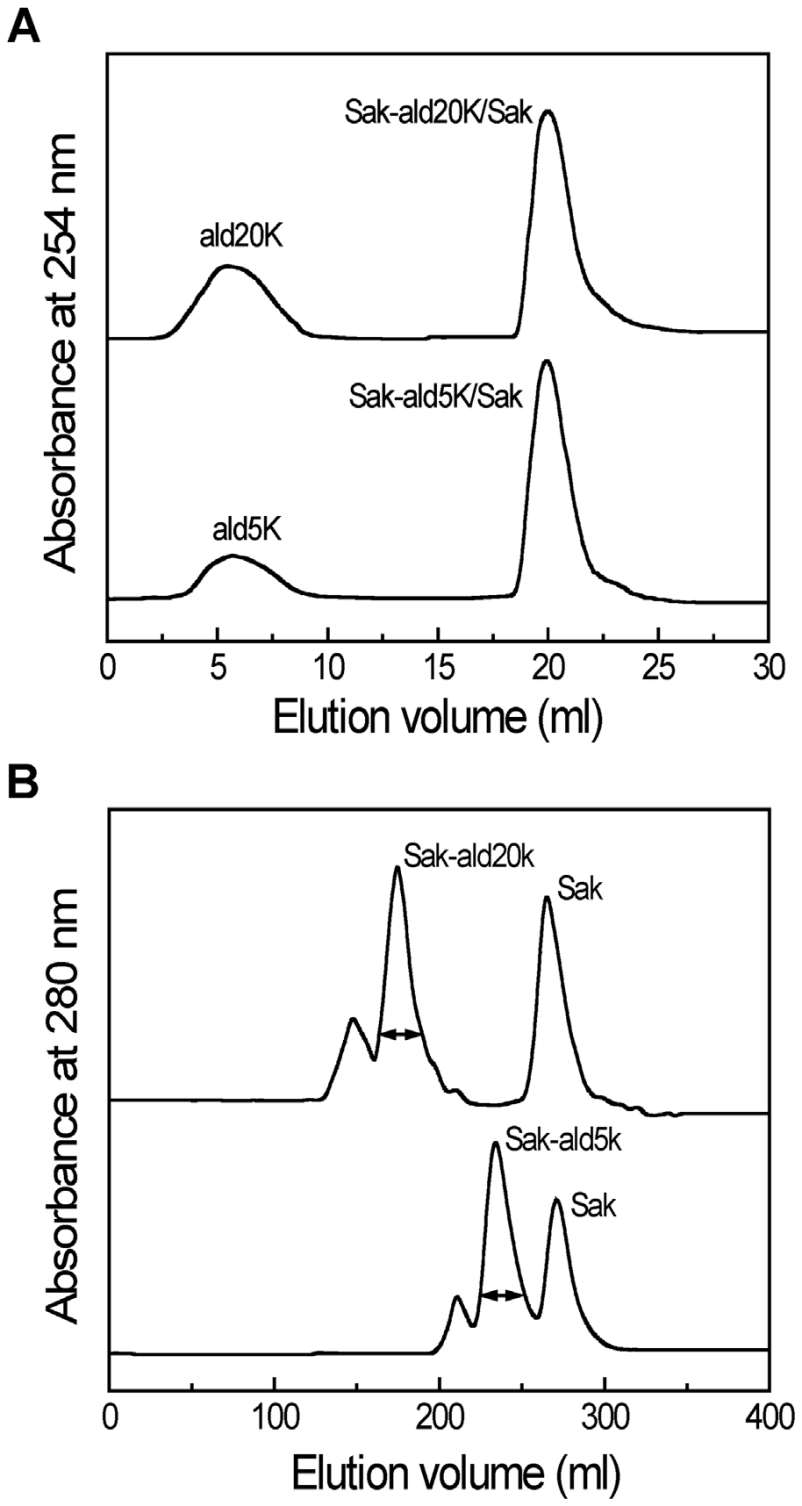
Purification of the PEGylated Sak by ald5k and ald20k. (A) The sample was loaded on an SP Sepharose HP column (16 mm ×25 mm). The column was equilibrated with 12 ml of 50 mM NaAc–HAc buffer (pH 5.0, Buffer A) and then with 18 ml 0.5 M NaCl in Buffer A at a flow rate of 1.0 ml/min. (B) The fractions corresponding to the proteins were loaded on a Superdex 200 column (2.6 cm ×60 cm) equilibrated and eluted with 20 mM sodium phosphate buffer (pH 7.2) at a flow rate of 3.0 ml/min.

Under the present experimental conditions, the PEGylated products by mal5k and mal20k were a mixture containing approximately 90% mono-PEGylated Sak and 10% unPEGylated Sak (data not shown). As shown in Fig. 2A, the free PEG was removed from the PEGylated products by ald5k and ald20k by the SP Speharose HP column. PEGylated products by ald5k and ald20k were a mixture containing 50–55% mono-PEGylated Sak, 10–15% highly PEGylated forms and 30–40% unPEGylated Sak (Fig. 2B). The mono-PEGylated Saks (Sak-ald5k and Sak-ald20k) were separated from the reaction mixtures by Superdex 200 column (2.6 cm×60 cm). The PEGylation site of Sak-ald5k and Sak-ald20k was characterized by tryptic peptide mapping according to Liu et al. [18] and demonstrated to be the N-terminus of Sak (data not shown).

**Figure 3:**
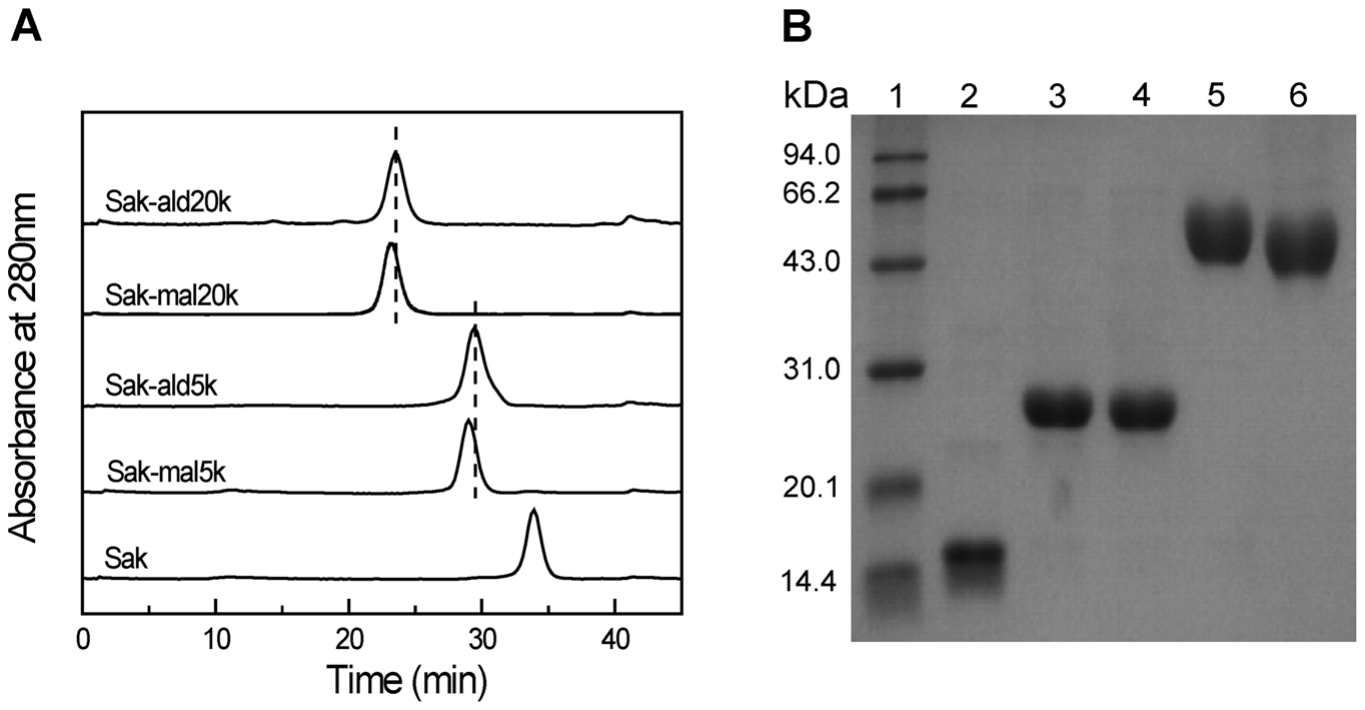
Characterization of the four PEGylated Saks. (A) SEC analysis was carried out on a Superdex 200 column (1 cm ×30 cm). The column was equilibrated and eluted with 20 mM sodium phosphate buffer (pH 7.2) at a ﬂow rate of 0.5 mL/min. (B) SDS-PAGE analysis of the samples. Lanes 1–6 were standard protein marker, Sak, Sak-mal5k, Sak-ald5k, Sak-mal20k and Sak-ald20k, respectively.

The purified PEGylated proteins were further analyzed by an analytical Superdex 200 column (1.0 cm×30 cm). As indicated by the SEC analysis (Fig. 3A), Sak-ald5k and Sak-mal5k are both eluted as single and symmetric peaks, which are left-shifted as compared with Sak. The elution peaks corresponding to Sak-ald20k and Sak-mal20k are further left-shifted as compared with Sak-ald5k and Sak-mal5k. This indicates that the hydrodynamic volume of Sak is enhanced by PEGylation and is dependent on the conjugated PEG mass. Moreover, the peak corresponding to Sak-mal5k is slightly left-shifted as compared to Sak-ald5k. Similarly, Sak-mal20k is eluted earlier than Sak-ald20k. This suggests that the PEGylation sites may alter the hydrodynamic volume of the PEGylated Sak.

As indicated by SDS-PAGE analysis (Fig. 3B), Sak shows a single electrophoresis band corresponding to an M_w_ of ∼15 kDa (Lane 2). As compared to Sak, Sak-ald5k (Lane 4) shows a single band with slower migration than Sak and slightly faster than Sak-mal5k (Lane 3). This indicates that the band migration of Sak is retarded by the conjugated PEG. Moreover, the PEGylation sites may determine the band migration of the PEGylated Sak. Similarly, Sak-ald20k (Lane 6) shows a single band with slower migration than Sak-ald5k and slightly faster than Sak-mal20k (Lane 5). Thus, SDS-PAGE analysis indicates the high purity of the four PEGylated Saks and further confirms the results of SEC analysis.

### Structural characterization of PEGylated Saks

**Figure 4:**
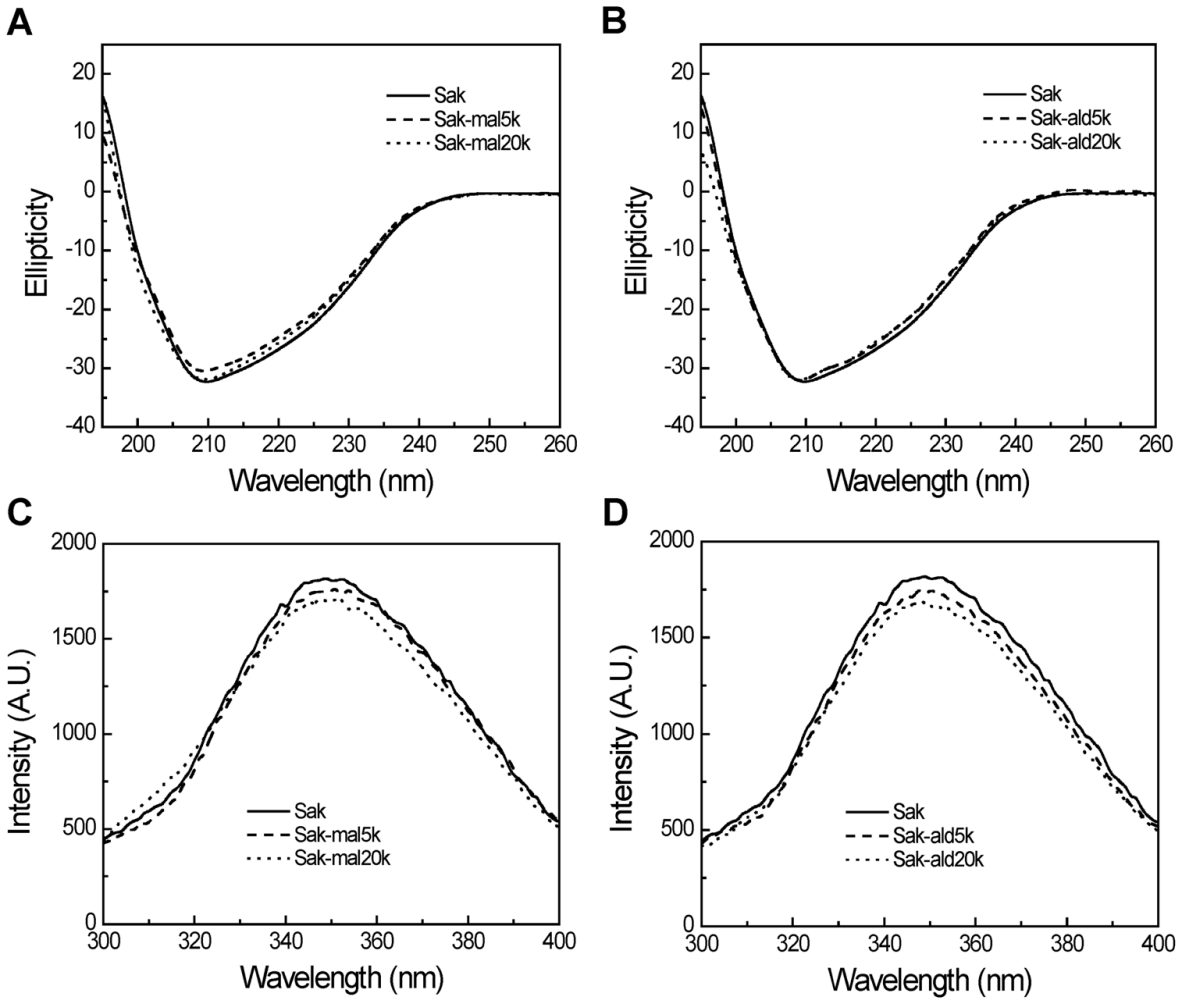
Structural characterization of the PEGylated Saks. Circular dichroism analysis was carried out for the two C-terminally PEGylated Saks (A) and the two N-terminally PEGylated Saks (B). Intrinsic fluorescence analysis was performed for the two C-terminally PEGylated Saks (C) and the two N-terminally PEGylated Saks (D).

CD analysis was used to investigate the secondary structure of Sak upon PEGylation. As shown in Fig. 4A and 4B, the far-UV CD spectra (200–260 nm) of Sak show a single band with a maximum at 208 nm, indicating the rich β-sheet of Sak. The CD spectra of the four PEGylated Saks are approximately superimposed on that of Sak. This indicates that the secondary structure of Sak is essentially not influenced by PEGylation.

Intrinsic fluorescence was applied to detect the conformational changes of Sak upon PEGylation. When excited at 280 nm, Sak shows maximum fluorescence intensity at 350 nm (Fig. 4 C, D). The emission fluorescence intensity of the four PEGylated Saks is comparable to that of Sak without shift in the maximum intensity at 350 nm. Therefore, PEGylation do essentially not perturb the conformation of Sak.

### Analytical ultracentrifugation

Sedimentation velocity analysis was performed to investigate the structure of the PEGylated Sak. The sedimentation coefficient (*S_20,w_*) and the ratio of frictional coefficient (*f/f_0_*) are summarized in Table 2. PEGylation can decrease the *S_20,w_* of Sak, due to the lower atom density of PEG chain relative to the protein core [29]. Moreover, Sak-mal20k and Sak-ald20k show lower *S^0^_20,w_* values than Sak-mal5k and Sak-ald5k. This indicates that the *S^0^_20,w_* of the PEGylated Sak is a function of the conjugated PEG mass. In addition, Sak-mal20k and Sak-mal5k show *S^0^_20_*_,*w*_ values comparable to those of Sak-ald20k and Sak-ald5k, respectively.

**Table 2:** Sedimentation velocity coefficients of the PEGylated Saks.

Sample	*S_20,w_* a	*f/f_0_* b
Sak	1.75±0.15	1.20
Sak-mal5k	1.36±0.05	1.48
Sak-ald5k	1.34±0.09	1.65
Sak-mal20k	1.21±0.04	2.35
Sak-ald20k	1.21±0.04	2.39

a The sedimentation coefficient *S* (10^−13^s) in a standard state of water at 20°C.

b The ratio of frictional coefficient.

The ratio of frictional coefficient (*f/f_0_*) is used to evaluate hydrodynamic shape of Sak [30]. Sak shows an *f/f_0_* of 1.20, indicating an almost spherical molecular shape. In contrast, PEGylation lead to the overall shape of Sak becoming geometrically asymmetric, as reflected by the increased *f/f_0_*. Moreover, the increase in *f/f_0_* is dependent on the PEG mass, indicating that the conjugated PEG may affect the hydrodynamic shape of Sak. In addition, Sak-mal20k and Sak-mal5k show lower *f/f_0_* values comparable to those of Sak-ald20k and Sak-ald5k, respectively. Thus, the PEGylation sites do affect the hydrodynamic shape of the PEGylated protein.

### *In vitro* bioactivity

**Figure 5:**
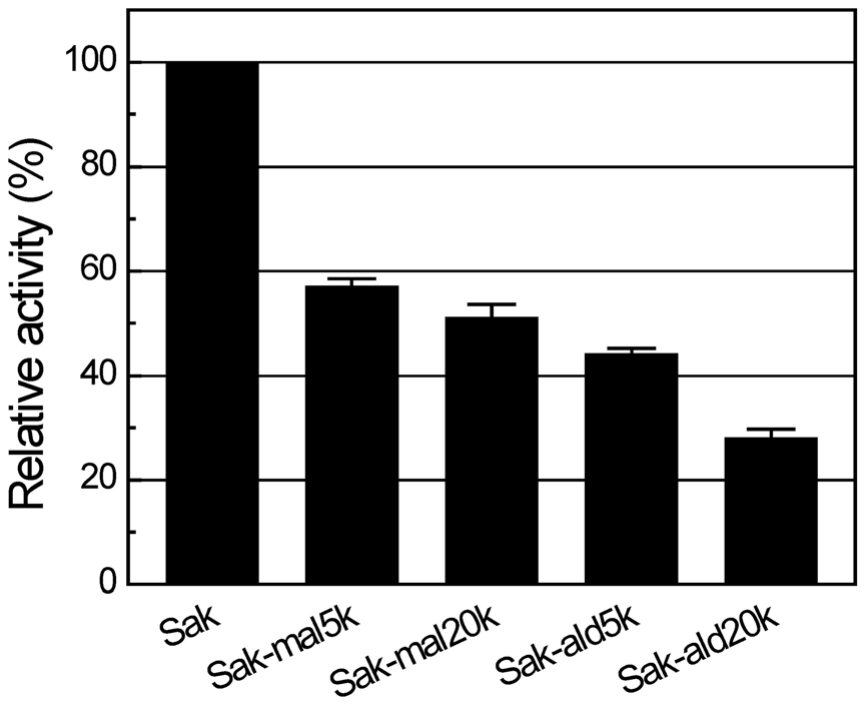
*In vitro* bioactivity of the PEGylated Saks. The *in vitro* bioactivity was tested by fibrin plate assay. The bioactivities of the PEGylated Saks were compared with that of the unmodified Sak, which was set to 100%.

The *in vitro* bioactivity of the products was measured by fibrin plate assay (Fig. 5). The relative bioactivities of the PEGylated Sak are lower than the unmodified Sak, presumably due to the steric shielding effect of PEG. Furthermore, Sak-mal20k and Sak-ald20k show lower bioactivities than Sak-mal5k and Sak-ald5k, respectively. This indicates that the steric shielding effect of PEG is dependent on the PEG mass. In addition, Sak-mal20k and Sak-mal5k show higher bioactivities than Sak-ald20k and Sak-ald5k, respectively. Presumably, N-terminus is close to the bioactive domain and C-terminus is far from it. Thus, the PEGylation site far from the bioactive domain may facilitate maintenance of the bioactivity of the PEGylated Sak. Atomic level investigation was conducted with the subsequent simulation research.

### Molecular dynamics simulation

#### Equilibration analyses

**Figure 6:**
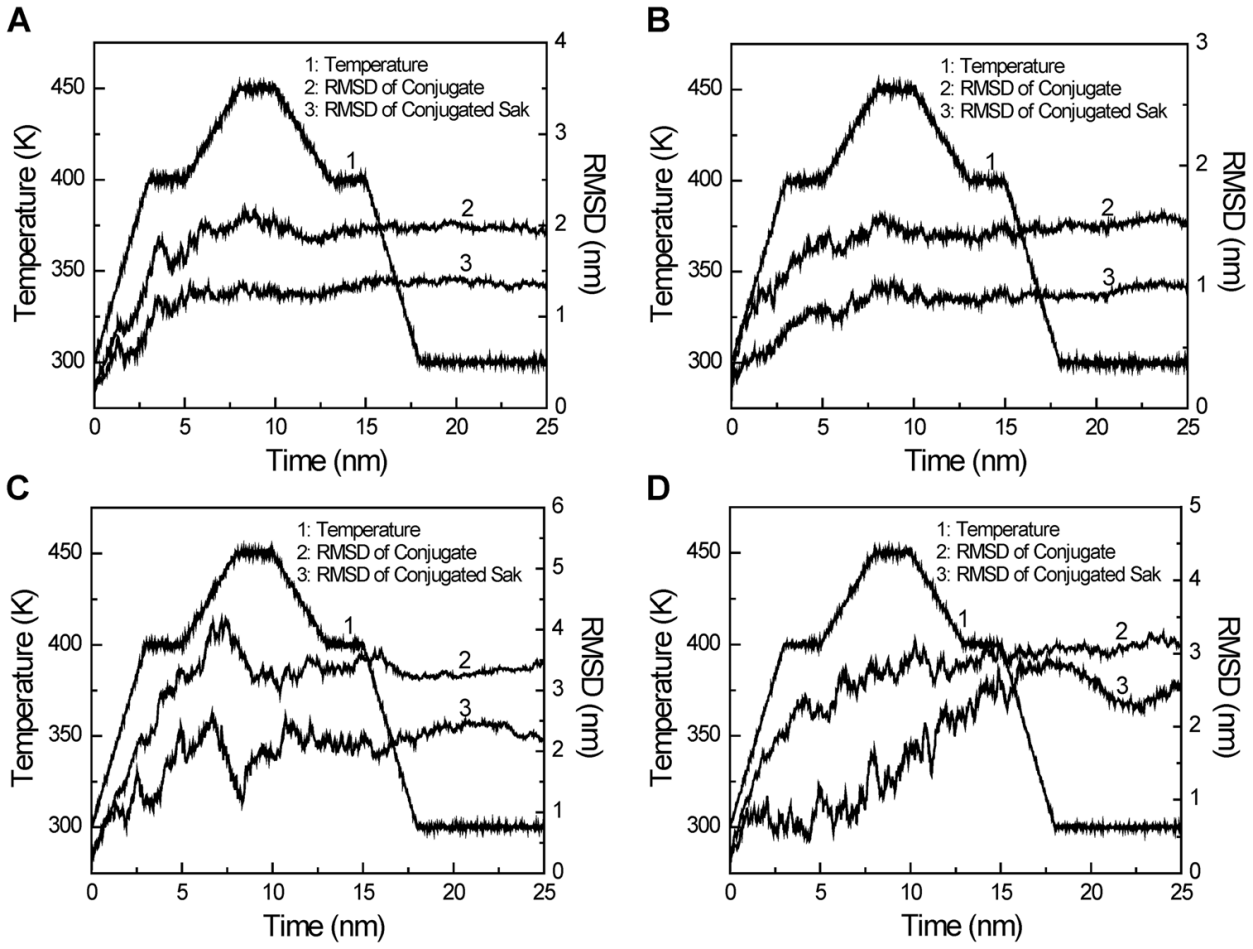
RMSDs of the PEGylated Saks during the simulated annealing period. (A) Sak-mal5k; (B) Sak-ald5k; (C) Sak-mal20k, (D) Sak-ald20k.

Fig. 6 shows that the PEGylated Sak system reaches the equilibrium state at the end of molecular dynamics simulation. In addition, the RMSDs (root mean square deviation) of Sak-mal20k and Sak-ald20k show larger fluctuations than those of Sak-mal5k and Sak-ald5k, indicating the movement of the flexible PEG chain. Fig. S6 shows snapshots of PEGylated Saks at different time points. Fig. S7 shows that Sak's secondary structures are apparently not altered with PEGylation.

**Figure 7:**
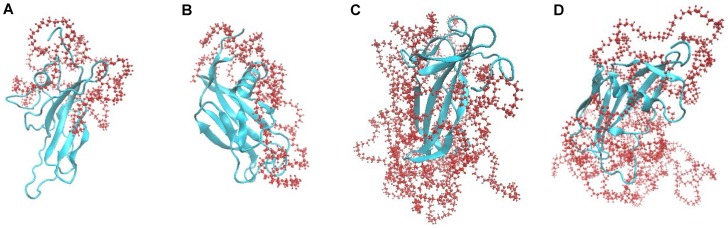
The conformations of PEGylated Saks after simulated annealing. (A) Sak-mal5k; (B) Sak-ald5k; (C) Sak-mal20k, (D) Sak-ald20k.

Fig. 7 shows that 5 kDa PEG chains partially cover the Sak surface (Fig. 7 A, B) while 20 kDa PEGs almost completely cover the Sak surface (Fig. 7 C, D). In addition, the PEG chain is not found to wrap around Sak tightly or with a regular pattern, rather it loosely folds on the surface of the protein in an irregular form.

#### Solvent accessible surface area (SASA) analyses

**Figure 8:**
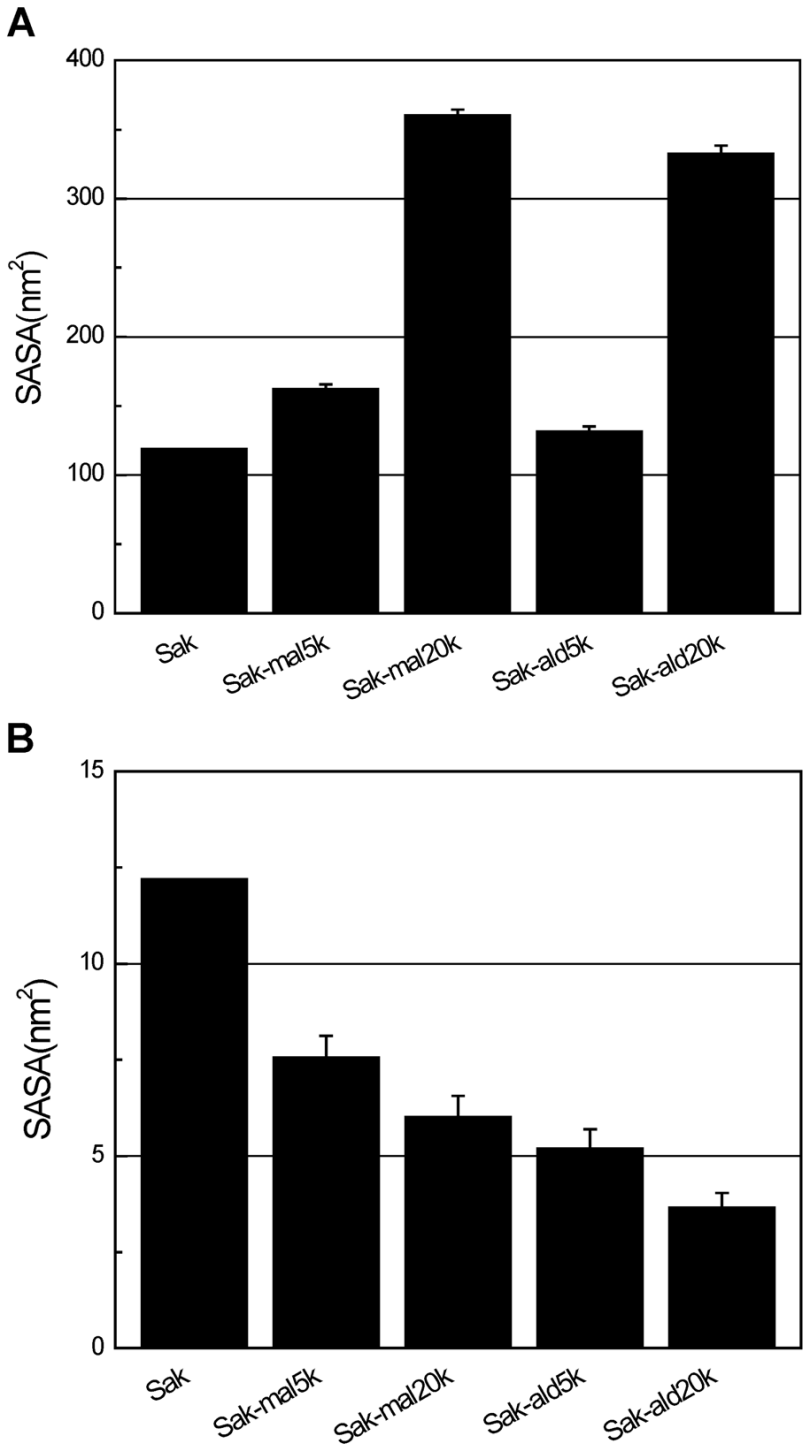
SASAs of the PEGylated Saks. Solvent accessible surface areas (SASA) were calculated by g_sas, a tool in GROMACS package. (A) Total SASAs of PEGylated products. (B) SASAs of the eight amino acids at Sak binding domain.

Computed SASA of different entities are shown in Fig. 8. SASA is calculated between 20 and 25 ns. Sak-mal5k and Sak-ald5k both show SASAs larger than Sak and much lower than Sak-mal20k and Sak-ald20k. This indicates that the PEG chain can induce a large hydrated layer. C-terminally PEGylated Saks show larger SASAs than the N-terminal ones with identical PEG mass (Fig. 8A), indicating a larger hydrodynamic volume of C-terminally PEGylated Sak.

To further investigate the steric shielding effect of PEG on the interaction of Sak and miro-plg, we computed SASA of the eight amino acids (Lys^11^, Asp^14^, Tyr^24^, Met^26^, Asn^28^, Tyr^44^, Glu^46^ and Ile^128^) in the binding domain of Sak [31]. Figure S8 shows active site SASA fluctuation from 20 to 25 ns. The PEGylated Saks show decreased SASAs compared with the free Sak (Fig. 8B), indicating the presence of the steric shielding effect of PEG. Moreover, SASAs of C-terminally PEGylated Saks (7.56 nm^2^ for Sak-mal5k and 6.02 nm^2^ for Sak-mal20k) are larger than that of N-terminal products (5.20 nm^2^ for Sak-ald5k and 3.67 nm^2^ for Sak-ald20k). Lower SASA of the eight amino acids indicates an intensified steric shielding effect of PEG, which would retard the binding ability of micro-plg to Sak. Computed SASA suggests that the bioactivity of Sak is decreased by the steric shielding effect of PEG. Moreover, the steric shielding effect of PEG conjugated at N-terminus is stronger than that at C-terminus, in spite of the larger hydrodynamic volume of C-terminally PEGylated Sak. Thus, computational result agrees well with results of *in vitro* bioactivity assay (Fig. 5).

#### Molecular size

Dynamic light scattering was used to measure the molecular radii of the PEGylated Saks. The radii of Sak, Sak-mal5k, Sak-ald5k, Sak-mal20k and Sak-ald20k are 2.77, 3.71, 3.34, 5.43 and 5.12 nm, respectively. The molecular volumes (V_e_) of the PEGylated Saks were thus calculated, assuming spherical shape of the PEGylated Saks (Table 3). The volumes at the end of simulation (V_s_) of PEGylated Saks were also calculated by rolling a probe with a radius of 1.4 Å over the molecular surface (Table 3). Clearly, V_s_ values of the PEGylated Saks are smaller than their corresponding V_e_ values. This is due to the fact that V_e_ of the PEGylated Sak consisted of the volumes from Sak, PEG itself and the hydrated layer of PEG, whereas V_s_ of the PEGylated Sak lacks the volume from the hydrated layer of PEG. V_e_ and V_s_ are found to be strongly correlated with each other (R^2^ = 0.9821), revealing the fact that MD simulation results correspond well to experimental data.

**Table 3:** Molecular volumes of the PEGylated products.

Sample	V_e_ (nm^3^) a	V_s_ (nm^3^) b
Sak	89	25.9
Sak-ald5k	156	33.8
Sak-mal5k	213.8	35.4
Sak-ald20k	562.1	65.8
Sak-mal20k	670.6	67.3

a V_e_: Molecular volumes measured by dynamic light scattering.

b V_s_: Molecular volumes calculated according to conformations of PEGylated Saks at the end of simulations.

#### Docking analyses

**Figure 9:**
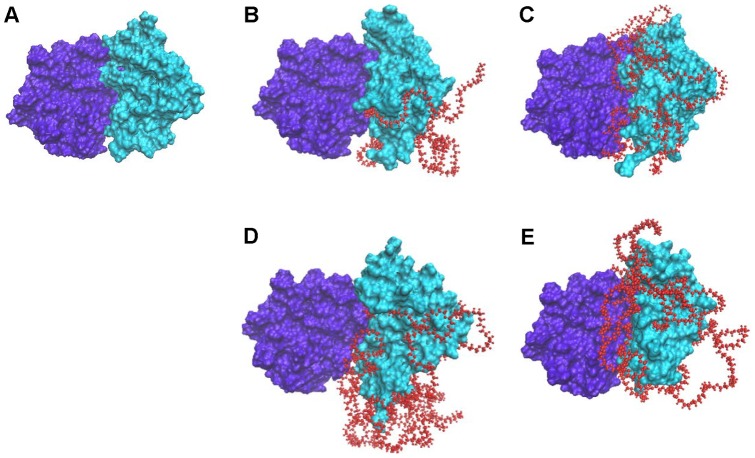
Final docked poses of PEGylated Saks and micro-plasminogen. (A) Free Sak, (B) Sak-mal5k; (C) Sak-ald5k; (D) Sak-mal20k; (E) Sak-ald20k.

Figure 9 shows final docked poses of PEGylated Saks and micro-plg. Table 4 shows the energy results of docking PEGylated Saks to micro-plg. The E-value represents the interaction energy between PEGylated Sak and its receptor (micro-plg). Lower E-value means higher stability of Sak-micro-plg complex and thus higher binding affinity of Sak to micro-plg. PEGylated Saks show higher E-values than free Sak, indicating that PEG hinders the binding of plasminogen to Sak. Moreover, Sak-mal5k and Sak-mal20k show lower E-values than Sak-ald5k and Sak-20k, respectively. This suggests that C-terminally PEGylated Saks show higher binding affinity to plasminogen than N-terminally PEGylated Saks, consistent to the experimentally assayed bioactivity of PEGylated Saks (Fig. 5).

**Table 4:** Docking results of PEGylated Saks with micro-Plasminogen.

Sample	E-value
Sak	−552.0
Sak-ald5k	−334.9
Sak-mal5k	−380.6
Sak-ald20k	−253.7
Sak-mal20k	−340.2

## Discussion

PEGylation of therapeutic proteins (e.g., cytokines) often leads to substantial loss of bioactivity, presumably due to the steric shielding effect of PEG which interferes with the interaction of the protein and its high M_w_ receptor. However, the steric shielding effect of PEG is not adequately understood so far, particularly at the molecular level. Our present study aimed to reveal the steric shielding effect of PEG on the therapeutic protein by experimental and molecular simulation analyses.

The steric shielding effect of PEG on Sak was investigated on the aspects of PEG chain length and PEGylation site. Accordingly, four PEGylated Saks were prepared by site-specific conjugation of 5 kDa/20 kDa PEG to the N-terminus and the C-terminus of Sak, respectively. Aldehyde chemistry was used for N-terminal PEGylation of Sak [32], [33] and site-specific PEGylation at the N-terminus was confirmed by tryptic peptide mapping analysis as described previously [18]. Maleimide chemistry was used for C-terminal PEGylation of Sak, which was achieved by PEGylation of the thiol group of the C-terminal Cys residue.

Structural characterizations suggest that the native conformation of Sak that underpins its bioactivity is essentially not altered upon PEGylation (Figs. 4 and S7). In contrast, the PEG chain length and the PEGylation site may alter its bioactivity (Fig. 5), hydrodynamic volume (Table 3) and molecular symmetry of the PEGylated Saks (Table 2).

Molecular dynamics simulation of the PEGylated Saks suggests that the PEG chain remains flexible at the equilibrium state. PEG can form a distinctive hydrated layer, which protects Sak, maintains its native conformation and increases its hydrodynamic volume. However, C-terminally PEGylated Saks show higher hydrodynamic volume than N-terminally PEGylated Saks, indicating higher flexibility of PEG in C-terminally PEGylated Sak that results in a larger hydrated layer. Presumably, the flexible PEG chain conjugated at the C-terminal domain of Sak is more loosely assembled on the Sak surface.

Simulation results (Fig. 7) show that PEG chain wraps around Sak and provides a steric shield around Sak. The steric shielding effect is dependent on PEG chain length and PEGylation site. Interestingly, the receptor binding domain of Sak is less sterically shielded by PEG in C-terminally PEGylated Sak, as reflected by the lower SASA of its receptor binding domain, which is presumably due to the fact that N-terminus is closer to the receptor binding domain of Sak than C-terminus. In addition, docking analyses indicate that stability of the Sak-micro-plg complex is inversely related to PEG chain length; stability is also found to be influenced by PEGylation site. These results further clarify the steric shielding effect of PEG on Sak and its effect on Sak interaction with its receptor.

Molecular sizes, both measured experimentally and computed from solvent accessible surfaces, had been found to correlate well with each other (R^2^ = 0.9821). Active site SASA and docking E-value were also found to correlate well with the relative bioactivity of Sak, with R^2^ = 0.9895 and R^2^ = 0.9937, respectively. They all point to the fact that molecular dynamics results and experimental data correlate with each other well. The above observations and cross-validation results show that molecular dynamics data has good predictive power (File S2).

## Conclusions

Wet lab assays and computational simulations were used to investigate the steric shielding effect of PEG on the conjugated Sak. Experimental study suggests that the native conformation of Sak is essentially not altered by PEGylation. In contrast, the bioactivity, the hydrodynamic volume and the molecular symmetric shape of the PEGylated Sak are altered and dependent on PEG chain length and PEGylation site. Molecular dynamics simulation of the PEGylated Saks suggests that the PEG chain remains highly flexible and forms a distinctive hydrated layer, resulting in the steric shielding effect of PEG on Sak. Docking analyses indicate that the binding affinity of Sak to its receptor also depends on PEG chain length and PEGylation site.

Computational simulation results agree well with experimental data. The present study provides clear molecular insight into the steric shielding effect of PEG on Sak at an atomic level. This type of study is essential to the rational design, fabrication and clinical application of PEGylated proteins, especially the ones involving interactions with high M_w_ substrates or receptors.

## Supporting Information

Figure S1 **Snapshots of PEG chain (10 units) during MD.** (A) Initial conformation: linear, force field: GROMOS_PEG; (B) Initial conformation: linear, force field: GROMOS_OE; (C) Initial conformation: coiled, force field: GROMOS_PEG; (D) Initial conformation: coiled, force field: GROMOS_OE.
(TIFF)

Figure S2 **Snapshots of PEG chain (20 units) during MD.** (A) Initial conformation: linear, force field: GROMOS_PEG; (B) Initial conformation: linear, force field: GROMOS_OE; (C) Initial conformation: coiled, force field: GROMOS_PEG; (D) Initial conformation: coiled, force field: GROMOS_OE.
(TIFF)

Figure S3 **Radii of gyration of PEG chains (10 units).** (A) Initial conformation: linear; (B) Initial conformation: coiled.
(TIFF)

Figure S4 **Radii of gyration of PEG chains (20 units).** (A) Initial conformation: linear; (B) Initial conformation: coiled.
(TIFF)

Figure S5 **PEG-Sak MD simulation from different initial PEG conformations.** (A) The radii of gyration of Sak-mal5k with different initial PEG conformations; (B) RMSDs between structures resulted from different initial conformations.
(TIFF)

Figure S6 **Snapshots of PEG-Saks during MD.** (A) Sak-mal5k; (B) Sak-ald5k; (C) Sak-mal20k; (D) Sak-ald20k.
(TIFF)

Figure S7 **Secondary structures of PEGylated Saks during MD.** (A) Sak-mal5k; (B) Sak-ald5k; (C) Sak-mal20k; (D) Sak-ald20k.
(TIFF)

Figure S8 **Active site SASAs of PEGylated-Saks at the equilibrium state.**
(TIFF)

File S1 **PEG parameterization in GROMOS 53a6 force field.**
(DOC)

File S2 **MD Cross-validation.**
(DOC)

## References

[1] Bailon P, Berthold W (1998) Polyethylene glycol-conjugated pharmaceutical proteins. Pharm Sci Technolo Today 1: 352–356.

[2] Milla P, Dosio F, Cattel L (2012) PEGylation of Proteins and Liposomes: a Powerful and Flexible Strategy to Improve the Drug Delivery. Curr Drug Metab 13: 105–119. 21892917 10.2174/138920012798356934

[3] Greenwald RB, Choe YH, McGuire J, Conover CD (2003) Effective drug delivery by PEGylated drug conjugates. Adv Drug Deliv Rev 55: 217–250. 12564978 10.1016/s0169-409x(02)00180-1

[4] Fishburn CS (2008) The pharmacology of PEGylation: Balancing PD with PK to generate novel therapeutics. J Pharm Sci 97: 4167–4183. 18200508 10.1002/jps.21278

[5] Zheng JC, Lei N, He QC, Hu W, Jin JG, et al (2012) PEGylation is effective in reducing immunogenicity, immunotoxicity, and hepatotoxicity of alpha-momorcharin in vivo. Immunopharmacol Immunotoxicol 34: 866–873. 22439816 10.3109/08923973.2012.666979

[6] Wattendorf U, Merkle HP (2008) PEGylation as a tool for the biomedical engineering of surface modified microparticles. J Pharm Sci 97: 4655–4669. 18306270 10.1002/jps.21350

[7] Tsutsumi Y, Onda M, Nagata S, Lee B, Kreitman RJ, et al (2000) Site-specific chemical modification with polyethylene glycol of recombinant immunotoxin anti-Tac(Fv)-PE38 (LMB-2) improves antitumor activity and reduces animal toxicity and immunogenicity. Proc Natl Acad Sci USA 97: 8548–8553. 10890891 10.1073/pnas.140210597 PMC26985

[8] Cazalis CS, Haller CA, Sease-Cargo L, Chaikof EL (2004) C-terminal site-specific PEGylation of a truncated thrombomodulin mutant with retention of full bioactivity. Bioconjug Chem 15: 1005–1009. 15366953 10.1021/bc049903y

[9] Dellacherie E, Leonard M (1991) Improvement of oxygen-carrying properties of human hemoglobin by chemical modification with a benzene hexacarboxylate-monosubstituted polyoxyethylene. J Protein Chem 10: 61–67. 2054064 10.1007/BF01024656

[10] Knusli C, Delgado C, Malik F, Domine M, Tejedor MC, et al (1992) Polyethylene-glycol (PEG) modification of granulocyte-macrophage colony stimulating factor (GM-CSF) enhances neutrophil priming activity but not colony stimulating activity. Br J Haematol 82: 654–663. 1482652 10.1111/j.1365-2141.1992.tb06940.x

[11] Chiu K, Agoubi LL, Lee I, Limpar MT, Lowe JW, et al (2010) Effects of polymer molecular weight on the size, activity, and stability of PEG-functionalized trypsin. Biomacromolecules 11: 3688–3692. 20979350 10.1021/bm1006954

[12] Tian P, Andricioaei I (2006) Size, motion, and function of the SecY translocon revealed by molecular dynamics simulations with virtual probes. Biophys J 90: 2718–2730. 16461399 10.1529/biophysj.105.073304 PMC1414555

[13] Lee HJ, Srinivasan D, Coomber D, Lane DP, Verma CS (2007) Modulation of the p53-MDM2 interaction by phosphorylation of Thr18: A computational study. Cell Cycle 6: 2604–2611. 17957142 10.4161/cc.6.21.4923

[14] Manjula BN, Tsai S, Upadhya R, Perumalsamy K, Smith PK, et al (2003) Site-specific PEGylation of hemoglobin at cys-93(beta): Correlation between the colligative properties of the PEGylated protein and the length of the conjugated PEG chain. Bioconjug Chem 14: 464–472. 12643758 10.1021/bc0200733

[15] Yang C, Lu DN, Liu Z (2011) How PEGylation enhances the stability and potency of insulin: A molecular dynamics simulation. Biochemistry 50: 2585–2593. 21332191 10.1021/bi101926u

[16] Collen D, Schlott B, Engelborghs Y, Vanhoef B, Hartmann M, et al (1993) On the mechanism of the activation of human plasminogen by recombinant staphylokinase. J Chem Biol 268: 8284–8289. 8463338

[17] Okada K, Ueshima S, Tanaka M, Fukao H, Matsuo O (2000) Analysis of plasminogen activation by the plasmin-staphylokinase complex in plasma of alpha(2)-antiplasmin-deficient mice. Blood Coagul Fibrinolysis 11: 645–655. 11085285 10.1097/00001721-200010000-00009

[18] Liu RY, Li DX, Wang J, Qiu R, Lin QX, et al (2012) Preparation, characterization and in vitro bioactivity of N-terminally PEGylated staphylokinase dimers. Process Biochem 47: 41–46.

[19] Ohlenschlager O, Ramachandran R, Guhrs KH, Schlott B, Brown LR (1998) Nuclear magnetic resonance solution structure of the plasminogen-activator protein staphylokinase. Biochemistry 37: 10635–10642. 9692953 10.1021/bi980673i

[20] Mark Thompson (2004) ArgusLab 4.0.1. Planaria Software LLC. Available: http://www.arguslab.com/arguslab.com/ArgusLab.html. Accessed 2012 Feb 16.

[21] Van der Spoel D, Lindahl E, Hess B, Groenhof G, Mark AE, et al (2005) GROMACS: Fast, flexible, and free. J Comput Chem 26: 1701–1718. 16211538 10.1002/jcc.20291

[22] Oostenbrink C, Villa A, Mark AE, van Gunsteren WF (2004) A biomolecular force field based on the free enthalpy of hydration and solvation: the GROMOS force-field parameter sets 53A5 and 53A6. J Comput Chem 25: 1656–1676. 15264259 10.1002/jcc.20090

[23] Frisch MJ, Trucks GW, Schlegel HB, Scuseria GE, Robb MA, et al.. (2009) Gaussian 09, Revision A.02, Gaussian, Inc., Wallingford CT.

[24] Winger M, de Vries AH, van Gunsteren WF (2009) Force-field dependence of the conformational properties of α,ω-dimethoxypolyethylene glycol. Molecular Physics 107: 1313–1321.

[25] Humphrey W, Dalke A, Schulten K (1996) VMD: Visual molecular dynamics. J Mol Graph 14: 33–38. 8744570 10.1016/0263-7855(96)00018-5

[26] Peisach E, Wang JY, de los Santos T, Reich E, Ringe D (1999) Crystal structure of the proenzyme domain of plasminogen. Biochemistry 38: 11180–11188. 10460175 10.1021/bi991130r

[27] Ritchie DW (2003) Evaluation of protein docking predictions using Hex 3.1 in CAPRI Rounds 1-2. Proteins 52: 98–106. 12784374 10.1002/prot.10379

[28] Parry MAA, Fernandez-Catalan C, Bergner A, Huber R, Hopfner KP, et al (1998) The ternary microplasmin-staphylokinase-microplasmin complex is a proteinase-cofactor-substrate complex in action. Nat Struct Biol 5: 917–923. 9783753 10.1038/2359

[29] Dhalluin C, Ross A, Leuthold LA, Foser S, Gsell B, et al (2005) Structural and biophysical characterization of the 40 kDa PEG-interferon-alpha(2a) and its individual positional isomers. Bioconjug Chem 16: 504–517. 15898716 10.1021/bc049781+

[30] Lebowitz J, Lewis MS, Schuck P (2002) Modern analytical ultracentrifugation in protein science: A tutorial review. Protein Sci 11: 2067–2079. 12192063 10.1110/ps.0207702 PMC2373601

[31] Jespers L, Vanwetswinkel S, Lijnen HR, Van Herzeele N, Van Hoef B, et al (1999) Structural and functional basis of plasminogen activation by staphylokinase. Thromb Haemost 81: 479–485. 10235424

[32] Hu T, Li D, Wang J, Wang Q, Liang Y, et al (2012) Propylbenzmethylation at Val-1(α) markedly increases the tetramer stability of the PEGylated hemoglobin: a comparison with propylation at Val-1(α). Biochim Biophys Acta 1820: 2044–2051. 23022153 10.1016/j.bbagen.2012.09.013

[33] Wang J, Hu T, Liu Y, Zhang G, Ma G, et al (2011) Kinetic and stoichiometric analysis of the modification process for N-terminal PEGylation of staphylokinase. Anal Biochem 412: 114–116. 21185800 10.1016/j.ab.2010.12.030

