# Supplementary figures and images for: Molecular Insight into the Steric Shielding Effect of PEG on the Conjugated Staphylokinase: Biochemical Characterization and Molecular Dynamics Simulation
Source: PLoS One. 2013 Jul 18;8(7):e68559. doi: 10.1371/journal.pone.0068559 (PMC3715476; doi:10.1371/journal.pone.0068559)

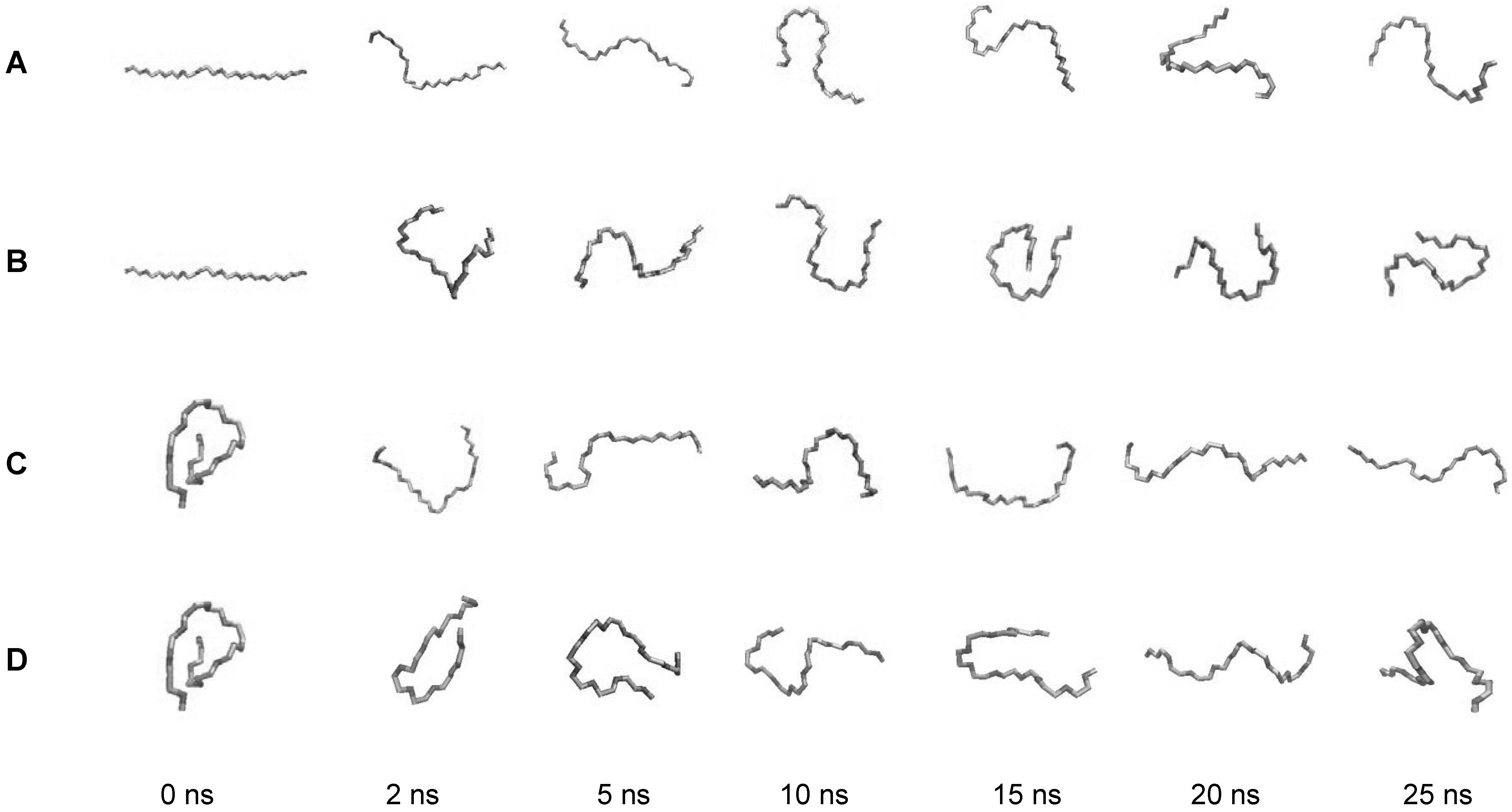

Supplement: Figure S1 — Snapshots of PEG chain (10 units) during MD. (A) Initial conformation: linear, force field: GROMOS_PEG; (B) Initial conformation: linear, force field: GROMOS_OE; (C) Initial conformation: coiled, force field: GROMOS_PEG; (D) Initial conformation: coiled, force field: GROMOS_OE. (TIFF) [file pone.0068559.s001.tiff]

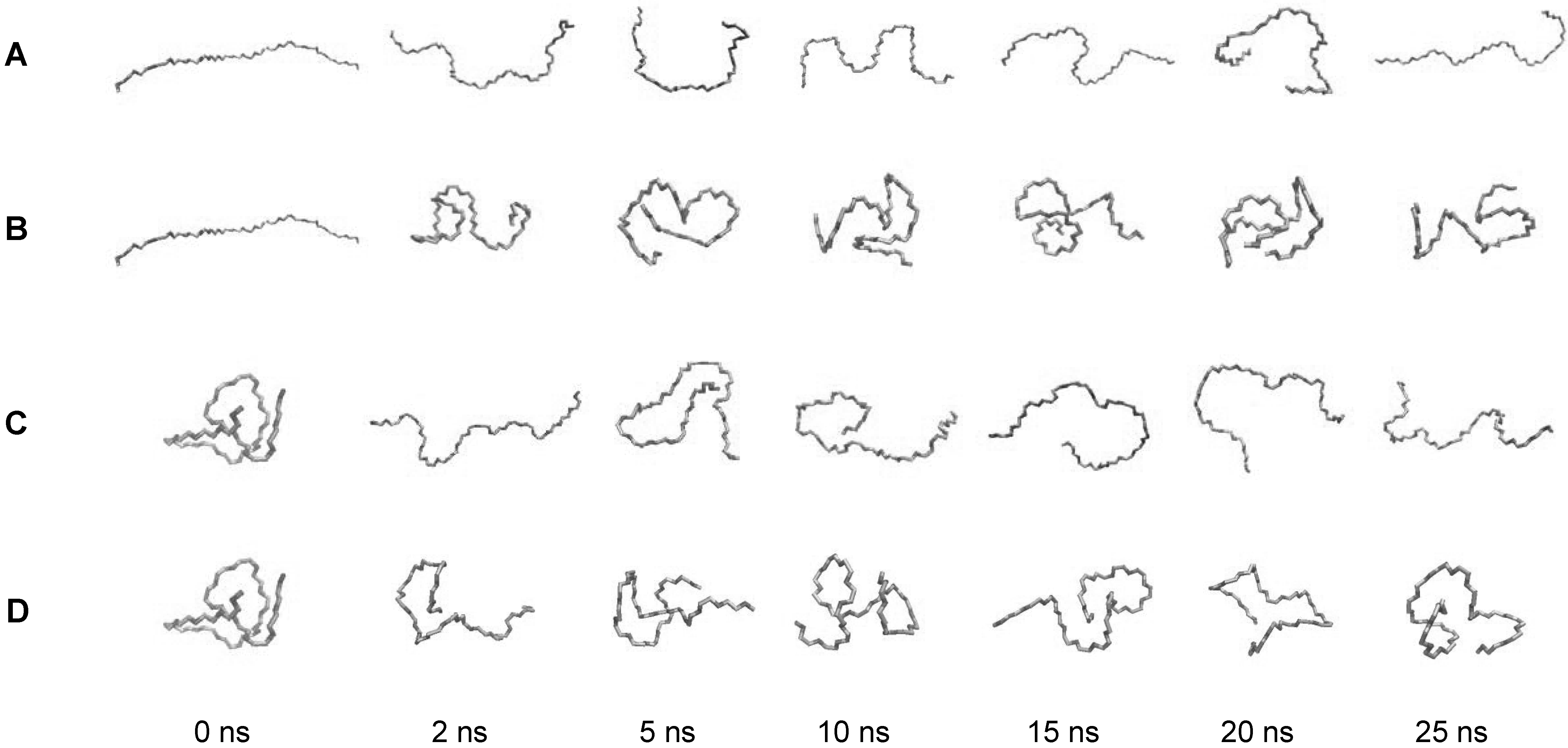

Supplement: Figure S2 — Snapshots of PEG chain (20 units) during MD. (A) Initial conformation: linear, force field: GROMOS_PEG; (B) Initial conformation: linear, force field: GROMOS_OE; (C) Initial conformation: coiled, force field: GROMOS_PEG; (D) Initial conformation: coiled, force field: GROMOS_OE. (TIFF) [file pone.0068559.s002.tiff]

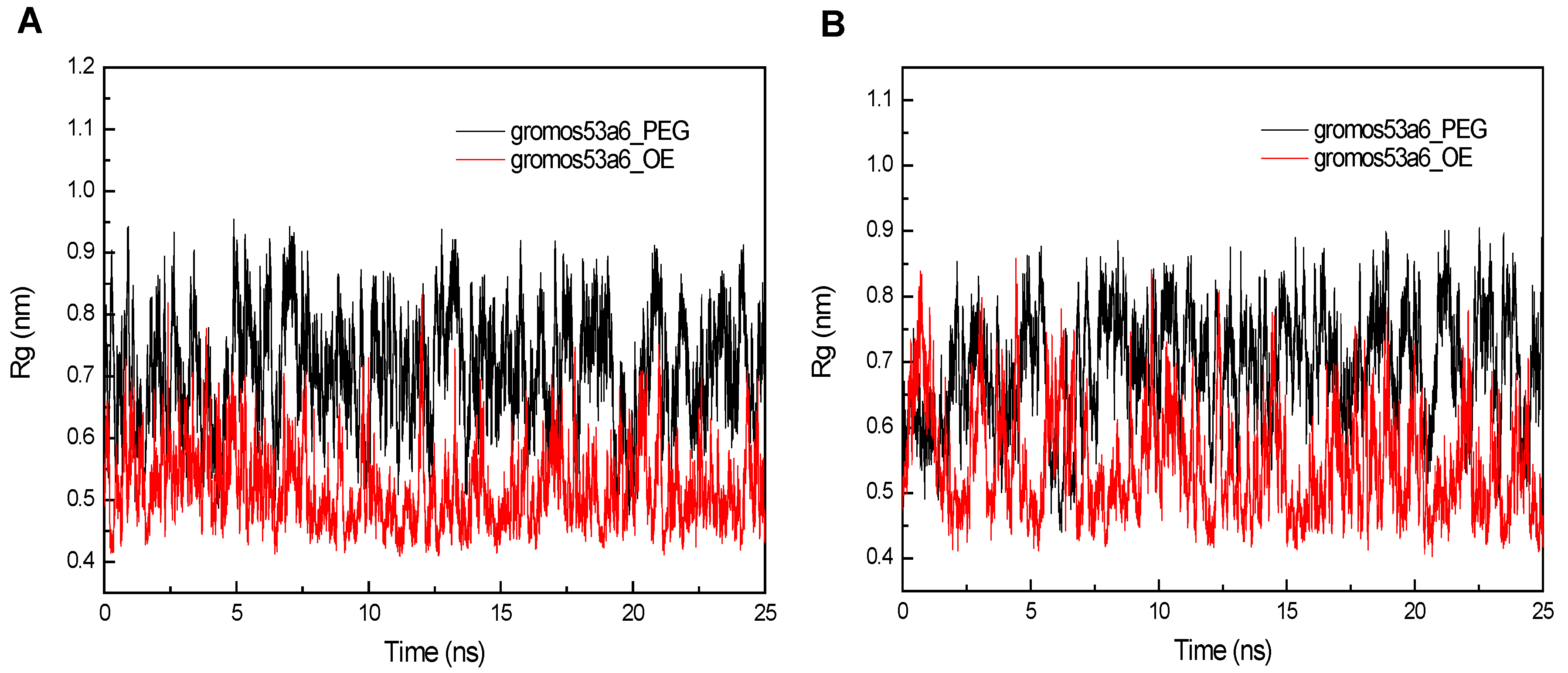

Supplement: Figure S3 — Radii of gyration of PEG chains (10 units). (A) Initial conformation: linear; (B) Initial conformation: coiled. (TIFF) [file pone.0068559.s003.tiff]

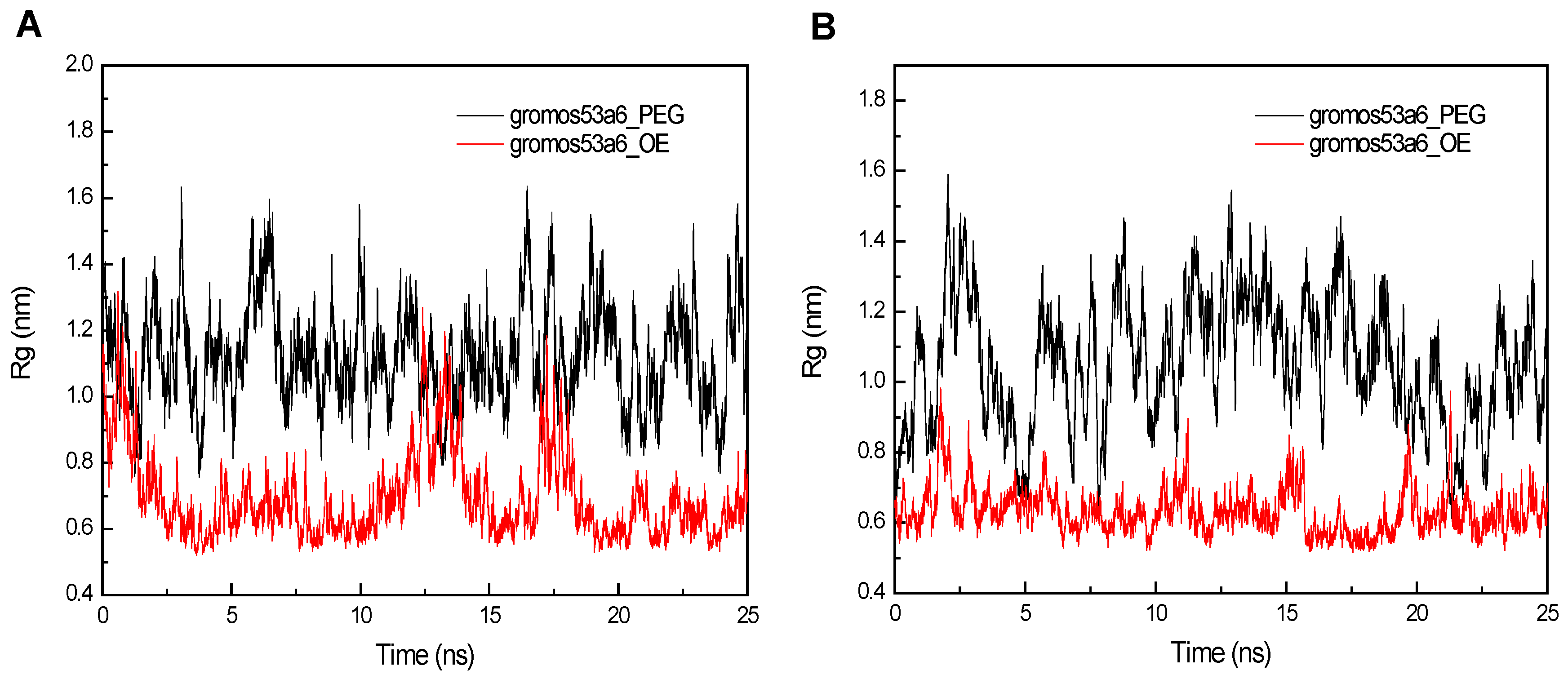

Supplement: Figure S4 — Radii of gyration of PEG chains (20 units). (A) Initial conformation: linear; (B) Initial conformation: coiled. (TIFF) [file pone.0068559.s004.tiff]

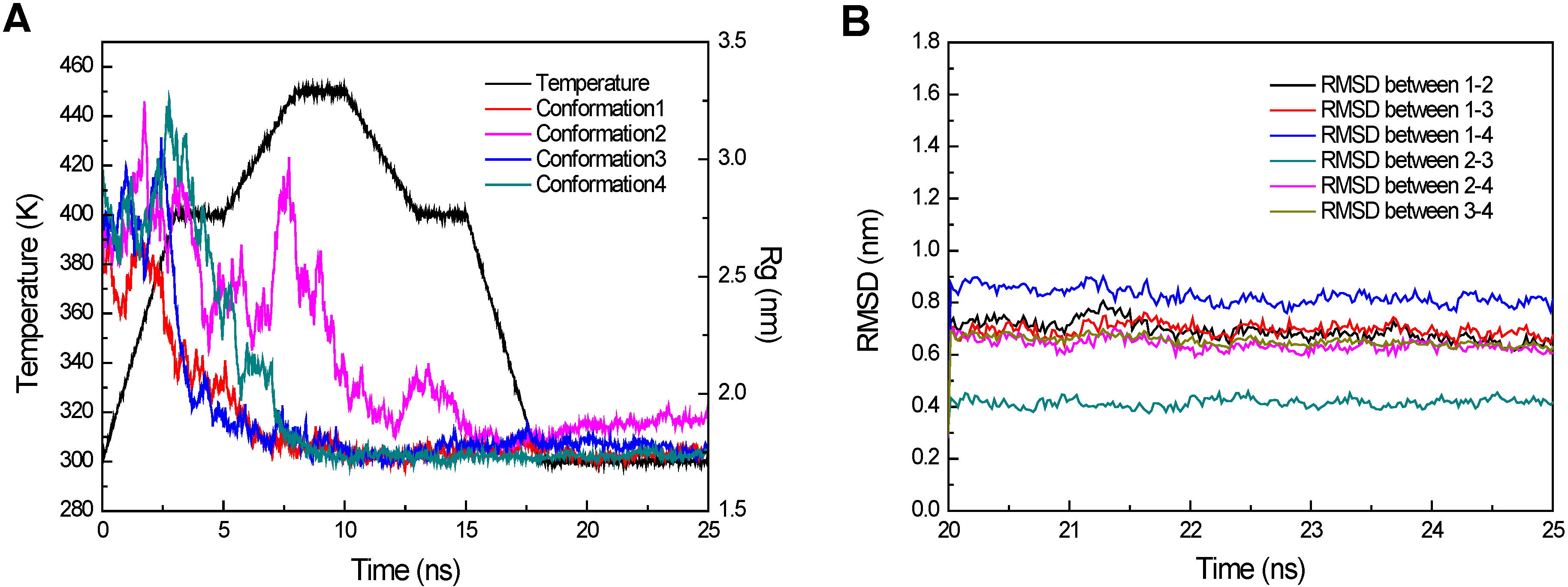

Supplement: Figure S5 — PEG-Sak MD simulation from different initial PEG conformations. (A) The radii of gyration of Sak-mal5k with different initial PEG conformations; (B) RMSDs between structures resulted from different initial conformations. (TIFF) [file pone.0068559.s005.tiff]

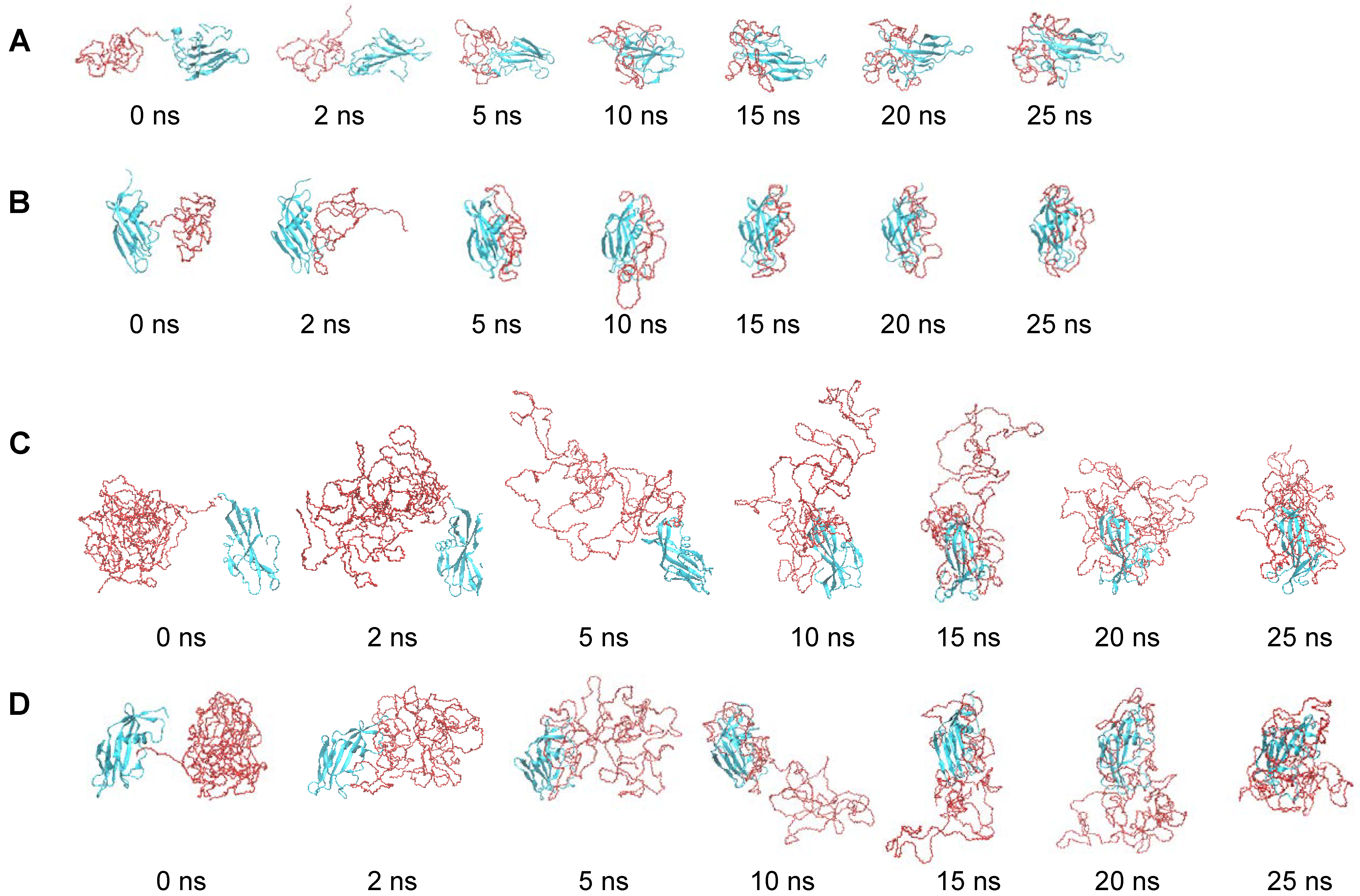

Supplement: Figure S6 — Snapshots of PEG-Saks during MD. (A) Sak-mal5k; (B) Sak-ald5k; (C) Sak-mal20k; (D) Sak-ald20k. (TIFF) [file pone.0068559.s006.tiff]

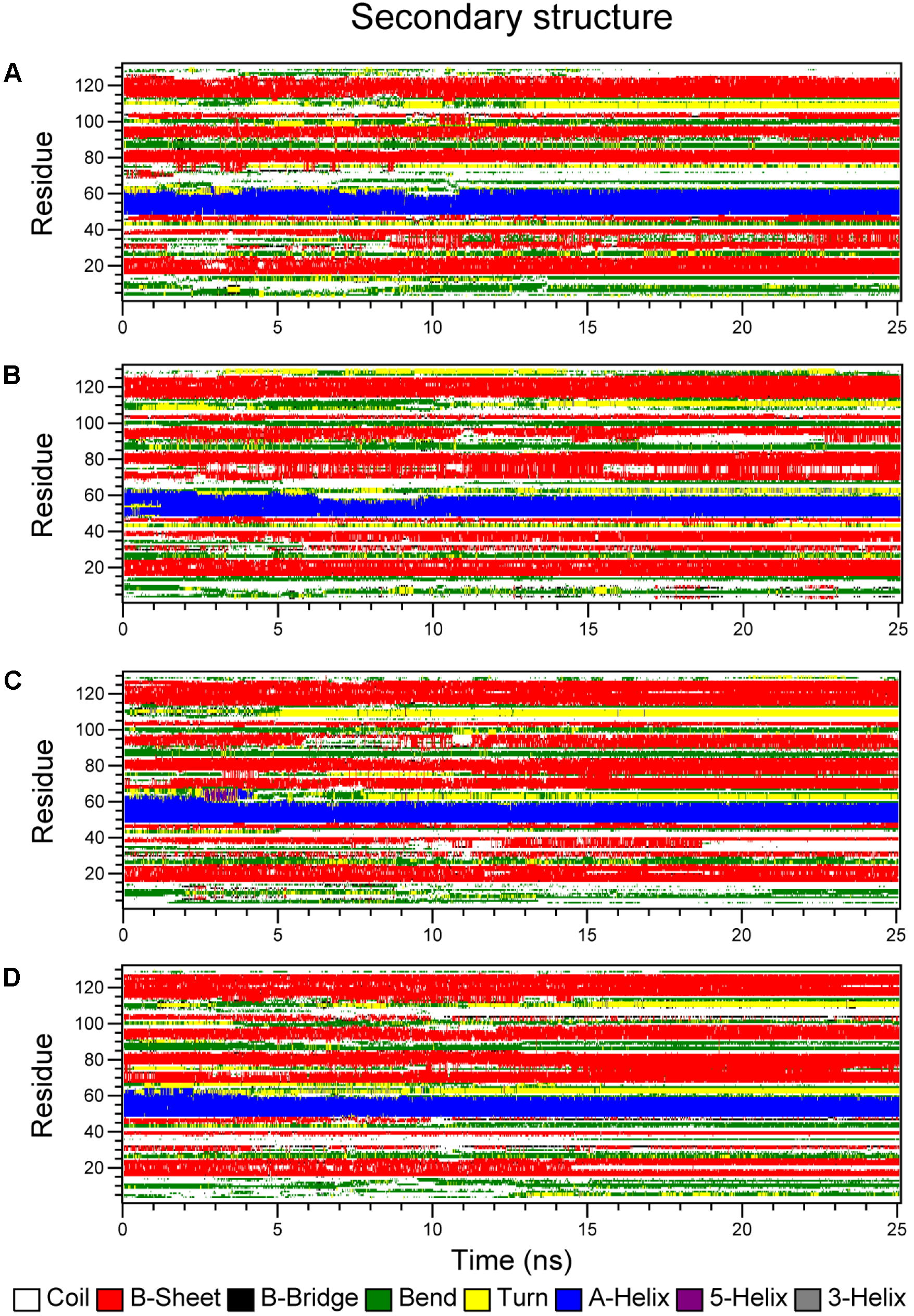

Supplement: Figure S7 — Secondary structures of PEGylated Saks during MD. (A) Sak-mal5k; (B) Sak-ald5k; (C) Sak-mal20k; (D) Sak-ald20k. (TIFF) [file pone.0068559.s007.tiff]

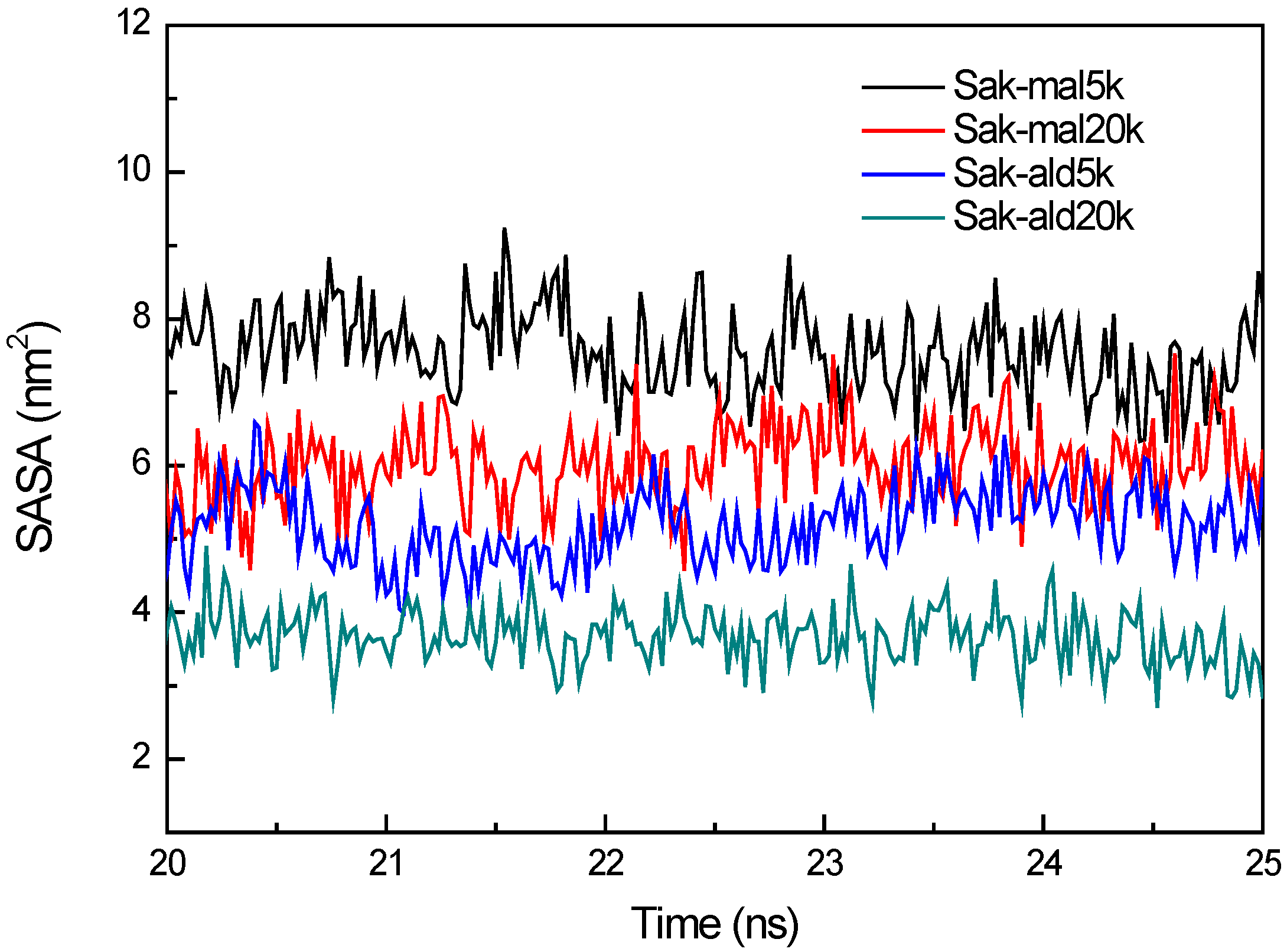

Supplement: Figure S8 — Active site SASAs of PEGylated-Saks at the equilibrium state. (TIFF) [file pone.0068559.s008.tiff]
