# Supplementary material for: Molecular Insight into the Steric Shielding Effect of PEG on the Conjugated Staphylokinase: Biochemical Characterization and Molecular Dynamics Simulation
Source: PLoS One. 2013 Jul 18;8(7):e68559. doi: 10.1371/journal.pone.0068559 (PMC3715476; doi:10.1371/journal.pone.0068559)
Supplement: File S1 — PEG parameterization in GROMOS 53a6 force field. (DOC) [file pone.0068559.s009.doc]

***PEG parameterization in GROMOS 53a6 force field***

GROMOS53a6 force field was developed for protein simulation. PEG parameters were derived and integrated into 53a6 to make it suitable for PEG-protein simulation. PEG middle unit (-CH_2_-O-CH_2_-) was defined as a residue named PEG while the terminal PEG unit (-CH2-O-CH3) was named PET. Partial charges of atoms in PEG and PET residues were determined through quantum computation in Gaussian 09W [1] using the Hartree-Fock algorithm. Bonded and non-bonded interaction parameters were not modified. The resulted force field was referred to as 53a6_PEG.

Winger *et al.* [2] also modified 53a6 to make it better suitable for PEG simulation, atom charge and Lennard-Jones interaction parameters were changed. The changed force field was called 53a6_OE.

53a6_PEG and 53a6_OE were compared by their performance in simulating short dimethoxypolyethylene glycol chains in water at 300 K. Different initial PEG states (linear or coiled) or different chain length (10 or 20 units) of PEG were used in simulation.

Figures S1 and S2 show that 53a6_PEG results in more extended PEG chains than 53a6_OE. Radii of gyration (Rg) of PEG chains are calculated and shown in figures S3 and S4. Rgs calculated from simulations of 53a6_PEG are larger than those from 53a6_OE, which indicates more loose PEG chains. The above observations point to the appropriateness of 53a6_PEG for PEG-protein simulation.

**References**

1. Frisch MJ, Trucks GW, Schlegel HB, Scuseria GE, Robb MA, et al. (2009) Gaussian 09, Revision A.02, Gaussian, Inc., Wallingford CT.
2. Winger M, de Vries AH, van Gunsteren WF (2009) Force-field dependence of the conformational properties of α,ω-dimethoxypolyethylene glycol. Molecular Physics 107: 1313-1321.
