# Supplementary material for: Molecular Insight into the Steric Shielding Effect of PEG on the Conjugated Staphylokinase: Biochemical Characterization and Molecular Dynamics Simulation
Source: PLoS One. 2013 Jul 18;8(7):e68559. doi: 10.1371/journal.pone.0068559 (PMC3715476; doi:10.1371/journal.pone.0068559)
Supplement: File S2 — MD Cross-validation. (DOC) [file pone.0068559.s010.doc]

***MD Cross-validation***

Cross-validation was performed as follows. Four PEGylation simulations (i.e., two different chains at two different sites) were performed in main text and final computed structures were docked to micro-plg. For Sak's active site SASAs, four pairs (SASA, relative bioactivity) were available. One of the pairs was withheld, the remaining three pairs, together with (SASA, relative bioactivity) for the free Sak, were fitted to a line, which was then used to compute relative bioactivity based on the withheld SASA. Differences between the measured and computed relative bioactivity were calculated and listed in Table A1, which shows differences less than 10%. Cross-validation for docking E-values was performed in the same fashion. Table A2 shows differences also less than 10%. Table A1 and A2 thus show that MD data do have predictive power.

**Tables**

**Table A1.** Cross-validation results for SASAs at Sak’s active site

| Sample | Measured relative bioactivity (A) | Relative bioactivity computed from SASA (B) | Difference  (C=∣A-B∣) | Deviation  (D=C/A) |
| --- | --- | --- | --- | --- |
| Sak-mal5k | 57 | 62.26 | 5.26 | 9.23% |
| Sak-ald5k | 44 | 41.03 | 2.97 | 6.75% |
| Sak-mal20k | 51 | 47.86 | 3.14 | 6.16% |
| Sak-ald20k | 28 | 30.35 | 2.35 | 8.39% |

**Table A2.** Cross-validation results for docking E-values

| Sample | Measured relative bioactivity (A) | Relative bioactivity computed from E-value (B) | Difference  (C=∣A-B∣) | Deviation  (D=C/A) | |
| --- | --- | --- | --- | --- | --- |
| Sak-mal5k | 57 | 58.29 | 1.29 | | 2.26% |
| Sak-ald5k | 44 | 47.79 | 3.79 | | 8.61% |
| Sak-mal20k | 51 | 47.44 | 3.56 | | 6.98% |
| Sak-ald20k | 28 | 26.54 | 1.46 | | 5.21% |
